# Ephrin-B2/Fc promotes proliferation and migration, and suppresses apoptosis in human umbilical vein endothelial cells

**DOI:** 10.18632/oncotarget.17298

**Published:** 2017-04-20

**Authors:** Li-Chun Zheng, Xiao-Qing Wang, Kun Lu, Xiao-Ling Deng, Cheng-Wei Zhang, Hong Luo, Xu-Dong Xu, Xiao-Man Chen, Lu Yan, Yi-Qing Wang, Song-Lin Shi

**Affiliations:** ^1^ Medical College of Xiamen University, Jinshan Community Health Service Center, Xiamen Traditional Chinese Medical Hospital, Xiamen 361000, P.R. China; ^2^ Xiamen Heart Center, Medical College of Xiamen University, Xiamen 361000, P.R. China; ^3^ Department of Basic Medicine, Medical College of Xiamen University, Cancer Research Center of Xiamen University, Xiamen 361102, P.R. China; ^4^ Department of Cardiology, Affiliated Dongnan Hospital of Xiamen University, Zhangzhou 363000, P.R. China; ^5^ Department of Basic Medicine, Medical College of Xiamen University, Xiamen 361102, P.R. China

**Keywords:** ephrin-B2, proliferation, migration, human umbilical vein endothelial cell, proteomic analysis

## Abstract

Tumor growth and metastasis are angiogenesis dependent. Angiogenic growth involves endothelial cell proliferation, migration, and invasion. Ephrin-B2 is a ligand for Eph receptor tyrosine kinases and is an important mediator in vascular endothelial growth factor-mediated angiogenesis. However, research offer controversial information regarding effects of ephrin-B2 on vascular endothelial cells. In this paper, proteome analyses showed that ephrin-B2/Fc significantly activates multiple signaling pathways related to cell proliferation, survival, and migration and suppresses apoptosis and cell death. Cytological experiments further confirm that ephrin-B2/Fc stimulates endothelial cell proliferation, triggers dose-dependent migration, and suppresses cell apoptosis. Results demonstrate that soluble dose-dependent ephrinB2 can promote proliferation and migration and inhibit apoptosis of human umbilical vein endothelial cells. These results also suggest that ephrinB2 prevents ischemic disease and can potentially be a new therapeutic target for treating angiogenesis-related diseases and tumors.

## INTRODUCTION

Tumor growth and metastasis are angiogenesis dependent. Blood vessels support cancer cells with nutrients and allow communication between primary and metastatic tumors [1–3]. Anti-angiogenesis is an efficient antitumor strategy for its protective effects against tumor emergence and progression [4–6]. Tyrosine protein kinase family includes ephrin and its receptor Eph as leading members and is considered as key factor for angiogenesis and lymphatic valve development [7, 8]. As one of the key genes in angiogenesis [9], ephrin-B2 is very important in regulating embryonic and adult angiogenesis and tumor angiogenesis [10–12].

Ephrin-B2 is variably expressed in tumor cells and mediates tumor cell proliferation, invasion, and migration [13, 14]. Through Src-extracellular-signal-regulated kinase (ERK) pathway, 5-fluorouracil-induced ephrin-B2 reverse signaling promotes tumorigenesis and drives epithelial–mesenchymal transition via Src-focal adhesion kinase (FAK) pathway [15]. Soluble ephrinB2 inhibits xenograft growth of head-and-neck squamous cell carcinoma [16]. Some studies showed that ephrin-B2 is a poor prognostic indicator of solid tumors, including head-and-neck squamous cell carcinoma, pancreatic adenocarcinoma, bladder urothelial carcinoma, and thyroid carcinoma [14, 17–19]

Vascular endothelial growth factor (VEGF) is the most potent cytokine involved in tumor angiogenesis and metastasis. VEGF-A binds to its receptor VEGFR2, induces VEGFR2 endocytosis, and promotes survival, proliferation, vessel sprouting, and permeability of endothelial cell vessels [12, 20, 21]. Ephrin-B2 was proven to be involved in VEGF/VEGFR-mediated angiogenesis. Ephrin-B2 promotes VEGFR endocytosis in endothelial cells, thereby enhancing VEGF-mediated angiogenesis [12, 22], which is essential in normal and pathological situations [23, 24].

Migration and proliferation of endothelial cells are prerequisites of solid tumor angiogenesis. However, research offer controversial information regarding effects of ephrin-B2 on vascular endothelial cells. Some studies showed that ephrin-B2 promotes migration and proliferation of endothelial cells [25–27]. On the other hand, other studies drew different conclusions [16, 28]. Systematic research is still needed to further clarify mechanisms of ephrin-B2 and its effects on endothelial cells.

In the present study, by using quantitative global and phosphorylated proteomics technological methods associated with bioinformatics analysis, we systematically and comprehensively revealed effects of ephrin-B2 on cellular functions and signaling pathways of human umbilical vein endothelial cells (HUVECs). Results showed that ephrin-B2 promoted proliferation, survival, migration, cell cycle, and suppressed apoptosis of HUVECs. Similar results were observed in further cellular experiments. These data suggest that ephrinB2 plays a protective role in ischemic disease and can potentially be a new therapeutic target for treating angiogenesis-related diseases and tumors.

## RESULTS

### Identification and quantification of differentially expressed proteins induced by ephrin-B2 treatment

To study the effect of ephrin-B2 on HUVECs, we isolated and identified primary HUVECs from freshly isolated umbilical cords. Immunohistochemical results demonstrated positive expression of factor-VIII-related antigen in isolated cells (Supplementary Figure 1); this positive expression proved successful isolation of HUVECs. HUVECs were cultured in stable isotope labeling by amino acids in cell culture (SILAC) media and treated with ephrin-B2/Fc or phosphate-buffered saline (PBS) for 15 h. Global and phosphorylated proteins were then prepared and subjected to liquid chromatography tandem-mass spectrometry analysis. Among 1720 global proteins identified, 258 were differentially expressed; 134 proteins increased, whereas 124 proteins decreased. For phosphorylated proteins, 395 differentially expressed proteins were identified from 1377 phosphorylated sites. According to phosphorylation level, 354 proteins were up-regulated, whereas 41 proteins were down-regulated.

### Functional analyses: Ephrin-B2 promoted proliferation and migration and suppressed apoptosis in HUVECs

**Figure 1:**
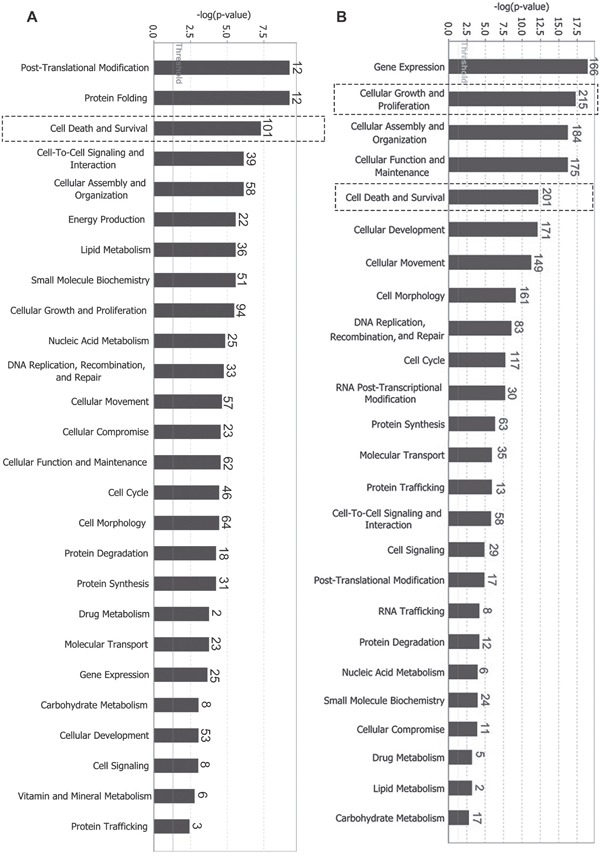
Differentially expressed proteins mainly participate in cell growth and proliferation, death and survival, cell movement, and other biological functions **(A)** Molecular and cellular functions involving differentially expressed global proteins; **(B)** Molecular and cellular functions involving differentially expressed phosphorylated proteins. *p*-value was calculated using right-tailed Fisher's exact test to assess association level. Functions were arranged according to -log(*p*-value). Threshold line indicating significant level was set at *p* = 0.05, or -log(*p*-value) = 1.3 means. The number on top of bar represents differentially expressed proteins involved in specific molecular pathways.

To functionally characterize differentially expressed proteins, we used Ingenuity Pathway Analysis (IPA) to associate these proteins to certain molecular and cellular functions in the Ingenuity Knowledge Base database. *p*-value was calculated using right-tailed Fisher's exact test to assess association level. Functions were arranged per -log(*p*-value). As shown in Figure 1A, for differentially expressed global proteins, the top five molecular and cellular functions were post-translational modification, protein folding, cell death and survival, cell-to-cell signaling and interaction, and cellular assembly and organization. For differentially expressed phosphorylated proteins, the top five molecular and cellular functions were gene expression, cellular growth and proliferation, cellular assembly and organization, cellular function and maintenance, and cell death and survival (Figure 1B). Among these functions, cell death and survival (labeled with dotted box) exhibited relatively high association levels and differential molecules in both global and phosphorylated proteins. Cellular growth and proliferation presented the highest number of differential molecules (215) in phosphorylated proteins. Z-scores were calculated according to expression level (up or down) of corresponding molecules in experiments and expected expression values (up or down) in pathways or functions. Z-scores ≥ 2 (≤ -2) indicate that function or signaling pathway is statistically and significantly activated (inhibited). As listed in the tables (Tables 1 and 2 and Supplementary Tables 3 and 4), with high absolute values of z-scores (|z-score| ≥ 2), ephrin-B2 suppressed cell apoptosis (z = -3.563/-2.329) and death (z = -3.5373/ -2.938) and promoted cell survival (z = 2.8823/3.825), proliferation (z = 2.425/3.818), movement (z = 4.2183/3.118), and migration (z = 3.9823/3.194). Ephrin-B2 also promoted angiogenesis (z = 2.928) in phosphorylated proteins (Supplementary Table 4).

**Table 1:** Significant biological functions associated with differentially expressed global proteins

Function Annotation	*p*-value	Predicted activation state	z-score
Apoptosis	1.51E-06	Inhibited	-3.563
Cell Death	4.97E-08	Inhibited	-3.5373
Necrosis	1.57E-05	Inhibited	-3.074
Cell Survival	6.26E-03	Activated	2.8823
Cell Movement	6.05E-05	Activated	4.2183
Migration of Cells	2.27E-05	Activated	3.9823
Proliferation of Epidermal Cells	7.32E-03	Activated	2.425
Reorganization of Cytoskeleton	7.53E-04	Activated	2.194
Organization of Cytoplasm	2.11E-04	Activated	2.036
Organization of Cytoskeleton	3.59E-04	Activated	2.074
Migration of Endothelial Cells	2.07E-03	Activated	2.013

Note: “Function annotation” includes biological functions involving differentially expressed proteins. *p*-value represents matching contingency of data, and corresponding function, |z-score| ≥ 2 is considered as prediction index of downstream effect.

**Table 2:** Significant biological functions associated with differentially expressed phosphorylated proteins

Diseases or Functions Annotation	*p*-Value	Predicted Activation State	z-score
Proliferation of Cells	5.43E-18	Activated	3.818
Pericardial Effusion	3.24E-03	Inhibited	-2.000
Cardiomyopathy	5.74E-04	Inhibited	-2.093
Development of Cardiovascular System	8.24E-05	Activated	2.398
Angiogenesis	2.81E-04	Activated	2.928
Development of Blood Vessel	1.67E-03	Activated	2.120
Interphase	7.37E-07	Activated	2.623
G1 Phase	1.97E-05	Activated	2.959
Cell Survival	2.76E-05	Activated	3.825
Cell Death	6.58E-13	Inhibited	-2.938
Apoptosis	7.07E-13	Inhibited	-2.329
Cell Viability	1.18E-04	Activated	3.916
Cell Movement	5.76E-12	Activated	3.118
Migration of Cells	2.04E-11	Activated	3.194
Edema	1.18E-04	Inhibited	-2.473
Repair of DNA	1.45E-08	Activated	2.163

### Signal pathway analyses: Ephrin-B2 activated signaling pathways involved in cell growth, proliferation, and movement

**Figure 2:**
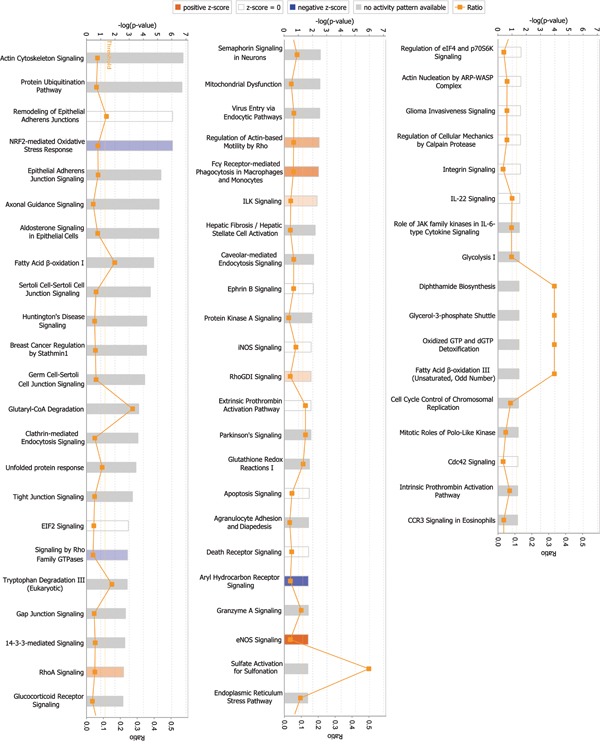
Signaling pathways associated with differentially expressed global proteins Among signaling pathways associated with differentially expressed global proteins, eNOS signaling exhibited the highest correlation and z-score (2.236), indicating pathway activation. *p*-value was calculated using right-tailed Fisher's exact test to assess association level. *p* < 0.05 or -log(*p*-value) > 1.3 (the main axis or bar chart) signifies significant association. Threshold line indicating significant level was set at *p* = 0.05 or -log(*p*-value) = 1.3 means. Z-score is calculated according to expression level (up or down) of corresponding molecules in experiments and expected expression value (up or down) in pathways. Z-scores ≥ 2 or ≤ -2 indicate that signaling pathway was statistically and significantly activated (colored in yellow) or inhibited (colored in blue). The ratio denotes proportion of differentially expressed genes in total signaling pathway genes; such ratio is provided as reference for significance assessment.

**Figure 3:**
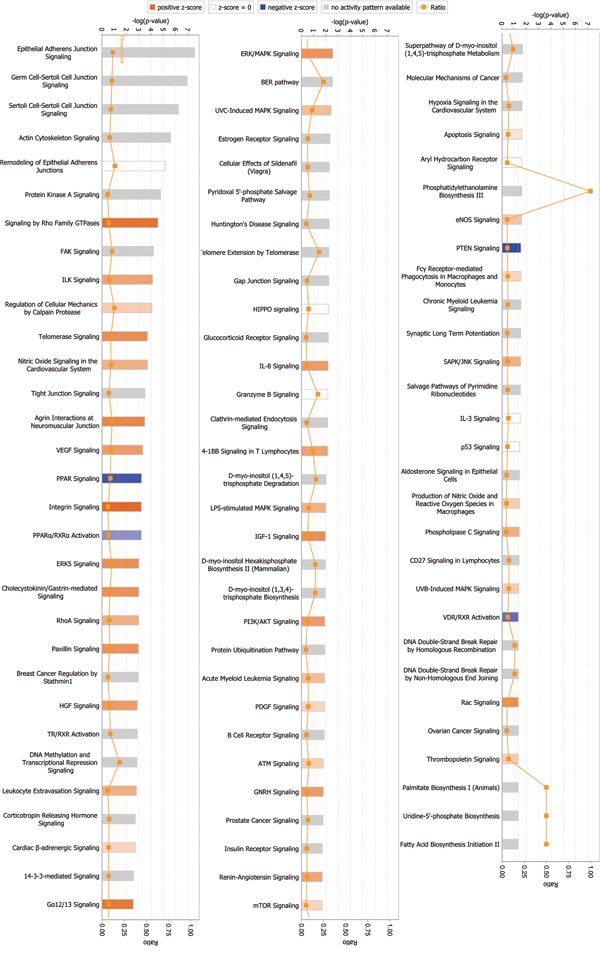
Signaling pathways associated with differentially expressed phosphorylated proteins Signaling by Rho GTPases exhibited the highest correlation among signaling pathways. *p*-value was calculated using right-tailed Fisher's exact test to assess association level. *p* < 0.05 or -log(*p*-value) > 1.3 (the main axis or bar chart) signifies significant association. Threshold line indicating significant level was set at *p* = 0.05, or -log(*p*-value) = 1.3 means. Z-score was calculated per expression level (up or down) of corresponding molecules in experiments and expected expression value (up or down) in pathways. Z-scores ≥ 2 or ≤ -2 indicate that signaling pathway was statistically and significantly activated (colored in yellow) or inhibited (colored in blue). The ratio denotes proportion of differently expressed genes in total signaling pathway genes. This ratio was provided as reference for significance assessment.

Among signaling pathways involving differentially expressed global proteins, endothelial nitric oxide synthase (eNOS) signaling showed the highest association level and z-score (2.236), indicating activation of this pathway (Figure 2, Table 3, and Supplementary Table 5). Rho GTPases showed the highest association level among signaling pathways involving differentially expressed phosphorylated proteins. Many important signaling pathways showed significant association and were activated in phosphorylated proteins (Figure 3, Table 4, and Supplementary Table 6). These signaling pathways include integrin signaling, GA12/13 signaling, telomerase signaling, ERK5 signaling, insulin-like growth factor 1 (IGF-1) signaling, Rac signaling, ERK/mitogen-activated protein kinase (MAPK) signaling, VEGF signaling, hepatocyte growth factor (HGF) signaling, and phosphoinositide-3-kinase (PI3K)/AKT signaling. These pathways are mainly involved in cell growth, proliferation, movement, and oxidative stress.

**Table 3:** Significant canonical pathways associated with differentially expressed global proteins

Ingenuity Canonical Pathways	-log(*p*-value)	Ratio	z-score
eNOS Signaling	1.68E00	3.55E-02	2.236
PPARα/RXRα Activation	1.3E00	2.79E-02	1.342
Regulation of Actin-based Motility by Rho	2.45E00	5.49E-02	1.000
RhoA Signaling	2.61E00	4.92E-02	0.816
NRF2-mediated Oxidative Stress Response	6.06E00	6.67E-02	-0.447

**Table 4:** Significant canonical pathways associated with differentially expressed phosphorylated proteins

Ingenuity Canonical Pathways	-log(*p*-value)	Ratio	z-score
Integrin Signaling	3.22E00	6.93E-02	3.464
Signaling by Rho Family GTPases	4.56E00	7.69E-02	3.153
Gα12/13 Signaling	2.56E00	7.69E-02	3.000
ERK5 Signaling	3.01E00	1.11E-01	2.449
IGF-1 Signaling	1.96E00	7.22E-02	2.449
Rac Signaling	1.33E00	5.77E-02	2.449
ERK/MAPK Signaling	2.56E00	6.42E-02	2.309
VEGF Signaling	3.33E00	9.89E-02	2.121
HGF Signaling	2.88E00	8.57E-02	2.121
PI3K/AKT Signaling	1.91E00	6.5E-02	2.121
IL-8 Signaling	2.18E00	6.01E-02	2.111

### Interaction network analyses indicated that ephrin-B2 participated in multiple cellular functions

To characterize the effects of ephrin-B2 on HUVECs by functional protein groups, identified differentially expressed proteins, called focus genes in IPA, were used as starting point to generate biological networks defined by the Ingenuity Knowledge Base database. Out of 258 differentially expressed global proteins, 79 were mapped to four highly significant networks (score ≥ 10, Supplementary Table 1 and Supplementary Figure 2), whereas out of 395 differentially expressed phosphorylated proteins, 131 were mapped to six highly significant networks (score ≥ 10, Supplementary Table 2 and Supplementary Figure 3). Networks were scored for likelihood of finding focus gene(s) in a given network. Biological relationship between two nodes was represented as an edge (line). Node color intensity indicated up-regulation (red) or down-regulation (green) degree. These networks demonstrated functional protein groups that are associated with multiple functions, such as cell death and survival, cellular assembly and organization, cellular growth and proliferation, cell cycle, cell morphology, and cellular movement, suggesting that ephrin-B2 is involved in multiple cellular functions.

### Results of *in vitro* cytological experiments

Proteomic analysis indicated that ephrin-B2 promoted HUVECs proliferation, survival, migration, and cell cycle. To verify these effects, cytological experiments were performed *in vitro*.

#### Ephrin-B2 promoted HUVECs proliferation

**Figure 4:**
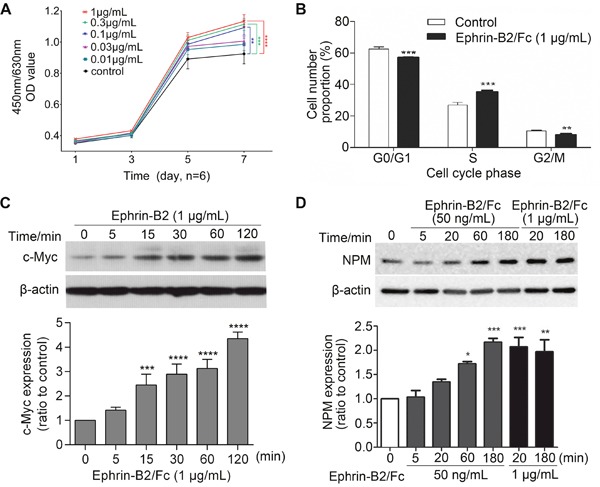
Ephrin-B2 promotes HUVECs proliferation **(A)** Ephrin-B2 significantly promoted endothelial cell proliferation at concentration of 0.3 or 1 μg/mL. Cell viability was detected using CCK-8 assay [mean ± SEM, n = 6, **p < 0.01, ***p < 0.001, ****p < 0.0001, by analysis of variance (ANOVA) and Dunnett's multiple comparison test]. **(B)** Effect of ephrin-B2 on cell cycle of HUVECs. Cells were treated with 1 μg/mL ephrin-B2/Fc for 24 h and then analyzed using cytometry (n = 3, mean ± SEM, **p < 0.01, ***p < 0.001, ****p < 0.0001 vs. control using Student's t-test). (**C** and **D**) Western blot showed the effect of ephrin-B2 on c-Myc and NPM protein expression levels. Bands were quantified using Quantity One software (mean ± SEM; ns, *p < 0.05, **p < 0.01, ***p < 0.001, and ****p < 0.0001 vs. control using ANOVA and Dunnett's multiple comparison test).

Cell Counting Kit-8 (CCK-8) assay showed that ephrin-B2 significantly promoted endothelial cell proliferation at concentration of 0.3 or 1 μg/mL (Figure 4A). Higher concentration allows faster cell growth, suggesting that ephrin-B2 enhanced HUVEC proliferation in dose-dependent manner. Cell cycle detection showed that G0/G1 phase cell ratio was significantly reduced after ephrin-B2 treatment, whereas S phase cell proportion was elevated (Figure 4B). Western blot results further indicated that expression levels of c-Myc and nucleophosmin (NPM), which promote cell proliferation, increased after ephrin-B2 treatment (Figure 4C and 4D).

#### Ephrin-B2 promoted HUVECs migration

**Figure 5:**
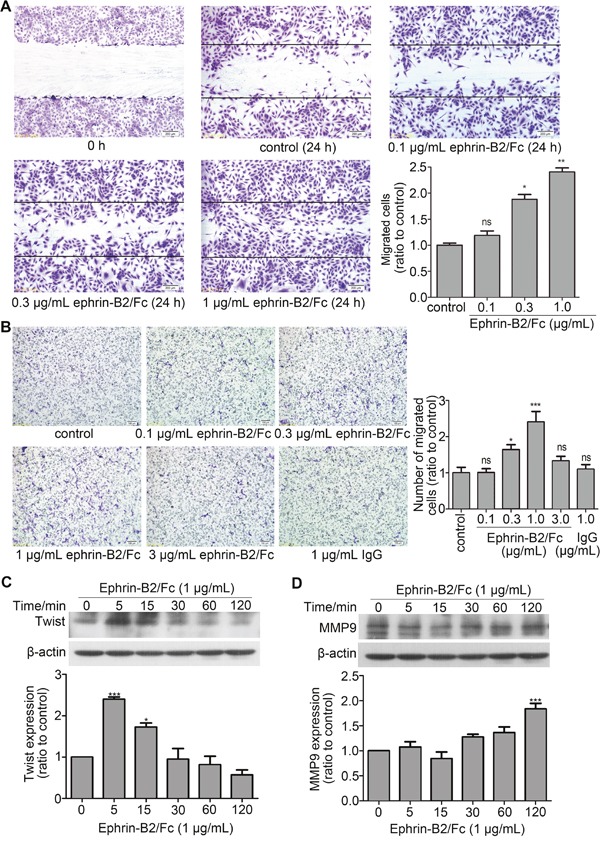
Ephrin-B2 promotes HUVEC migration **(A)** Wound-healing assay. Consistently shaped wounds were created after 12 h of serum starvation. Cells were cultured for 24 h in serum-free medium containing different ephrin-B2/Fc concentrations or PBS, fixed by 4% paraformaldehyde, and stained using crystal violet. Images were captured under the microscope (100 times magnification), and in each well, six different scraped areas were randomly selected and analyzed. Cells treated by 0.3 or 1 μg/mL ephrin-B2 showed higher migration ratio (n = 3, mean ± SEM; ns, *p < 0.05, **p < 0.01 vs. control using ANOVA and Dunnett's multiple comparison test). **(B)** Transwell assay. A total of 35,000 cells were cultured on the upper chamber using serum-free medium containing different ephrin-B2/Fc concentrations (0, 0.1, 0.3, 1, and 3 μg/mL), which were poured onto the bottom chamber as migration stimulator. Six hours later, migrated cells were fixed, stained, photographed, and then counted according to normal methods. Cells dose-dependently migrated under low concentrations (n = 3, mean ± SEM; ns, *p < 0.05 and ***p < 0.001 vs. control using ANOVA and Dunnett's multiple comparison test). **(C** and **D)** Western blot showed the effects of ephrin-B2 on Twist and MMP9 protein expression levels. Bands were quantified using Quantity One software (mean ± SEM; ns, *p < 0.05 and ***p < 0.001 vs. control using ANOVA and Dunnett's multiple comparison test).

Angiogenesis is mainly involved in endothelial cell migration and proliferation. Proteomic analysis indicated that ephrin-B2 can promote endothelial cell movement and migration. Wound healing experiment and Transwell assay were performed to confirm above results. Wound healing experiments showed that migration distance increased with ephrin-B2 concentration compared with that of the control group (Figure 5A). This result indicated dose-dependent enhancement of the effect of ephrin-B2 on HUVEC migration. Transwell experiment showed that ephrin-B2 promoted endothelial cell migration at concentrations from 0.1 μg/mL to 1 μg/mL and enhanced effect peaked at 1 μg/mL. However, number of migrated cells treated with 3 μg/mL ephrin-B2 did not significantly change compared with that of the control group (Figure 5B). This result indicated that the effect of ephrin-B2 on endothelial cell migration was dose-dependent at 0–1 μg/mL, and that enhanced effect disappeared when ephrin-B2 concentration reached 3 μg/mL. Western blot results further indicated that pro-migration protein Twist and matrix metallopeptidase 9 (MMP9) expression increased after treatment with 1 μg/mL ephrin-B2. Twist expression increased immediately to a high level (5 min) and then declined, whereas no significant increase was observed for MMP9 until 2 h after ephrin-B2 incubation (Figure 5C and 5D).

#### Ephrin-B2 inhibited apoptosis in HUVECs

**Figure 6:**
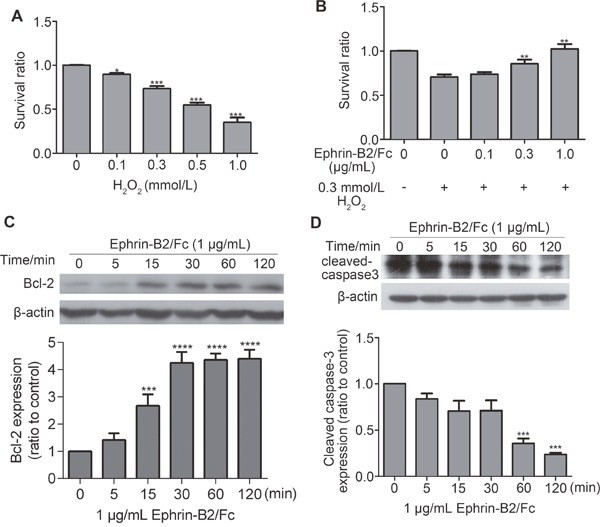
Ephrin-B2 inhibits HUVEC apoptosis **(A)** Cell apoptosis assay by CCK-8. Cell apoptosis was induced 4 h after incubation with different concentrations of H_2_O_2_. Cell survival ratios were then tested at OD450 nm. Relatively significant level of apoptosis was observed at concentration of 0.3 mmol/L (n = 3, mean ± SEM; ns, *p < 0.05, ***p < 0.001 vs. control using ANOVA and Dunnett's multiple comparison test). **(B)** Different concentrations of ephrin-B2/Fc (0.1, 0.3, and 1 μg/mL) contribute to protecting HUVECs from apoptosis, which is induced by H_2_O_2_ (0.3 mmol/L). Cells were cultured in serum-free medium containing different concentrations of ephrin-B2/Fc or PBS for 2 h then incubated with H_2_O_2_ for 4 h. Cell survival ratios were also tested on OD450 nm. Cells treated by 1 μg/mL ephrin-B2 showed higher survival ratio (n = 3, mean ± SEM; ns, **p < 0.01 vs. control using ANOVA and Dunnett's multiple comparison test). **(C**–**D)** Western blot showed the effects of ephrin-B2 on Bcl-2 and cleaved caspase 3 protein expression levels. Bands were quantified using Quantity One software (mean ± SEM; ns, **p < 0.01, ***p < 0.001 and ****p < 0.0001vs. control using ANOVA and Dunnett's multiple comparison test).

To detect cell apoptosis protection by ephrin-B2, H_2_O_2_ was used as inducer for apoptosis. As shown in Figure 6A, HUVECs survival ratio declined significantly when H_2_O_2_ concentration reached 0.3 mmol/L. After ephrin-B2 treatment for prior protection, survival ratio showed dose-dependent improvement (Figure 6B). Western blot analysis showed increased level of anti-apoptotic protein Bcl-2 expression after ephrin-B2 treatment (Figure 6C), whereas cleaved caspase 3 gradually decreased by 1 μg/mL ephrin-B2 (Figure 6D).

#### Ephrin-B2 activated some HUVEC signaling pathways

**Figure 7:**
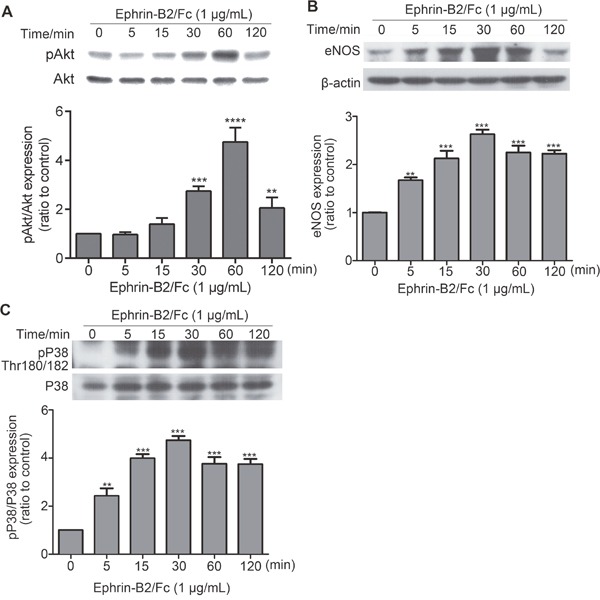
Effects of ephrin-B2 on signaling pathways in HUVECs HUVECs were treated with 1 μg/mL ephrin-B2 for 5, 15, and 30 min and for 1 and 2 h. Expression levels of pAkt **(A)**, eNOS **(B)**, and pP38 **(C)** were detected by Western blot. Intensity of each band was quantified by Quantity One software (mean ± SEM; ns, **p < 0. 01 and ***p < 0.001, ****p < 0.0001, vs. control using ANOVA and Dunnett's multiple comparison test). β-actin was used as internal control.

As shown in Figure 3, many signaling pathways were activated or inactivated by ephrin-B2. However, verifying all changes was difficult. We attempted to detect the effect of ephrin-B2 on PI3K-Akt, VEGF, and P38 signaling pathways, which have close relationship with cell proliferation, migration, and survival, respectively. As shown in Figure 7A, pAkt (Thr308) expression was increased by ephrin-B2. eNOS is a PI3K-Akt downstream factor; its expression level increased following Akt activation (Figure 7B). Ephrin-B2 treatment significantly increased pP38 (Thr180/182) level, demonstrating activation of P38 signaling pathway (Figure 7C).

## DISCUSSION

Endothelial cell proliferation and migration are two major events during angiogenesis [29]. Previous studies on the role of ephrin-B2 in endothelial cells mainly focused on cell growth, proliferation, and migration [25, 30]. In the present study, proteomic analysis revealed that ephrin-B2 significantly activated multiple signaling pathways related to cell proliferation, survival, and migration and suppressed apoptosis and nuclear factor erythroid 2-related factor 2 (NRF2)-mediated oxidative stress response. Cytological experiments further confirmed that ephrin-B2 stimulated proliferation, caused HUVECs dose-dependent migration, increased anti-apoptotic Bcl-2 expression, pro-proliferation proteins c-Myc and NPM, and pro-migration proteins Twist and MMP9, activated Akt/eNOS and P38 signaling pathways, and inhibited activation of caspase 3.

Different studies reported contradicting results regarding the effects of ephrin-B2 on endothelial cells. Some evidence indicated that by interfering VEGF and Ang-1 signal transmissions, ephrin-B2 inhibits proliferation, migration, and vascular budding of endothelial cells, where EphB4 receptors are located, thereby inhibiting angiogenesis [28, 30, 31]. By contrast, other studies showed that ephrin-B2 promotes proliferation and migration by activating EphB4 receptors [25, 32, 33]. This discrepancy may be explained by different experimental models used or ephrin-B2 concentration variations in those studies. In the current study, as exhibited in our cytological experiments, potency of promoting migration by ephrin-B2 was strong when concentration was set to 0.3 and 1 μg/mL, which are lower than the dosage used in previous studies. Endothelial progenitor cells were treated with 3 μg/ml Eph-B2-Fc in Foubert's study [34]. In Mansson-Broberg's study [35], 0.5, 5, and 10 μg/mL Eph-B2 were used in human adult endothelial cell cultures, and a single dose of 20 μg ephrinB2-Fc was used for C57BL/6 mice weighing 25 g. However, enhancement of migration by 3 μg/mL ephrin-B2 was not significant in the current study. Ephrin-B2 from different sources may possess different activity, possibly resulting in significant differences in effective concentration of ephrin-B2. However, variable results in previous reports and our study most possibly reflect the dependence of ephrin-B2 effects on experimental models or ephrin-B2 concentration used.

Previous studies explored specific signaling pathways to explain the effects of ephrin-B2 on cells. Such research is often segmentary, and results may not reflect all changes induced by ephrin-B2. By quantitatively analyzing alteration in global and phosphorylated protein expression profiles of HUVECs, the current study systematically and comprehensively uncovered cellular function changes and associated signaling pathways mediated by ephrin-B2. Previous studies on ephrin-B2 did not report alterations in these signaling pathways or their cellular functions. These alterations can provide clues to future research on ephrin-B2 mechanism. For example, inactivated signaling pathways including NRF2-mediated oxidative stress response, peroxisome proliferator activated-receptor (PPAR) signaling, and PPARα/retinoid X receptor α (RXRα) activation, were all related to oxidative stress (blue bar in Figure 2 and 3); though not meeting threshold z-score, these results may be a clue to explain Foubert's finding [34], which indicated that in wound healing, Eph-B2 improves beneficial effects of endothelial progenitor cell in physiological and irradiated conditions only when in association with antioxidant treatment. Mansson-Broberg's study [35] also showed that in hypoxic environment, proliferation rate of human adult endothelial cells is significantly lower in ambient air, and adding ephrin-B2 (0.05, 0.5, 5, and 10 μg/mL) increases proliferation rate (p < 0.10). These previous studies and ours revealed that inactivated oxidative stress related signaling pathways possibly modulate effects of ephrin-B2. The link between these phenomena is worth further study.

Here, we also observed an alteration on signaling pathway of Fc gamma receptor-mediated phagocytosis in macrophages and monocytes (Figure 2 and Figure 3). Although the z-score indicated by IPA is no of significance (1.342 in Figure 2 and 0.816 in Figure 3), there is still a possibility that part of response we detected may be due to Fc gamma receptor stimulation, since ephrin-B2/Fc was used as the stimulant. However, we do not think Fc gamma receptor cause too much disturbance to our conclusion. Recombinant protein ephrin-B2/Fc has been used by many researchers for studying the function of ephrin-B2 [36–38]. In fact, since half-life of Fc recombinant proteins is much longer than their original form, Fc has been widely used to produce Fc fusion proteins in pharmaceutical field [39]. Moreover, the only Fc receptor expressed in endothelial cells is the neonatal Fc receptor (FcRn) [40]. Its main function in endothelial cells is to protect IgG or fusion proteins from being metabolized [41]. Therefore, we think Fc is unlikely to cause any remarkable response in HUVECs.

In the present study, through a series of proteomic and cytological experiments, we proved that ephrin-B2 exerts dose-dependent effects on HUVEC proliferation and migration and suppresses cell apoptosis. Considering its positive roles in promoting endothelial cell proliferation and migration, ephrin-B2 is expected to be a therapeutic candidate in angiogenesis-related diseases and tumor.

As a reversible and transient modification, phosphorylation is mostly studied at the beginning stage of stimulation. In this study, we choose to perform analyzing events on changes of global and phosphorylated proteins at the same stage (15 h after stimulation), to make our conclusions more persuasive. We think that phosphorylation must play a key role in both the initial signal transduction and subsequent signaling pathway and protein function regulation. Phosphorylation is the most ubiquitous post-translational modification (PTM) a protein experiences throughout its lifetime. It serves to modify protein function by altering protein stability, cellular location, substrate affinity, complex formation, and activity [42]. Therefore, not only the expression level of proteins but also the phosphorylation level of related proteins is changed by ephrin-B2 at 15h. In fact, our results demonstrated a large number of phosphorylated proteins were changed at 15h (1377 phosphorylated sites of 395 proteins). And many biofunctions revealed by proteomics analysis of global and phosphorylated proteins were consistent (Table 1 and 2).

Notably, this current study examined only the effects of ephrin-B2 on HUVECs *in vitro*. Though results of proteomic analysis agree with results of cell-level assays, the present study cannot provide final answers to existing open questions or solve previous controversies. Further experiment *in vivo* should be carried out to clarify functions and mechanisms of ephrin-B2 on endothelial cell proliferation and migration.

## MATERIALS AND METHODS

### Chemical

Ephrin-B2/Fc was purchased from R&D Systems (USA). All antibodies were purchased from Cell Signaling Technology, Inc. SILAC kits were purchased from Thermo Fisher Scientific, Inc. (USA). Other chemical compounds were purchased from Sigma (USA).

### HUVEC isolation and culture

All research involving human subjects were conducted in agreement with the Declaration of Helsinki. Studies involving HUVECs were approved by the Ethical Review Board of the Medical College of Xiamen University, and an informed consent was signed by suppliers. Freshly isolated umbilical cords were obtained from affiliated Zhongshan Hospital of Xiamen University.

Umbilical veins were washed thrice with cold sterile PBS (Gibco). Both ends of umbilical veins were bound and filled with 0.05% trypsin-ethylenediaminetetraacetic acid (EDTA) buffer (Gibco). After incubation for 10 min at 37 °C, endothelial cells were washed with Dulbecco's modified Eagle's medium (DMEM) containing 20% fetal bovine serum (FBS, Gibco) and subsequently centrifuged at 400 × g for 10 min at 4 °C. Separated cells were cultured in DMEM containing 20% FBS, and adherent cells were preserved for passage in accordance with conventional method. In the present study, all cells used were cultured for four to six passages.

### SILAC labeling and cell treatment

Isolated HUVECs were cultured in DMEM medium containing 10% FBS at 37 °C with 95% air and 5% CO_2_. Cells were labeled with “heavy” SILAC media (Thermo) containing 0.1 g/L ^13^C_6_^15^N_2_L-Lysine-2HCl and 0.1 g/L ^13^C_6_^15^N_4_L-Arginine-HCl or “light” SILAC media (Thermo) containing 0.1 g/L L-Lysine-2HCl and 0.1 g/L L-Arginine-HCl. After five times of passage and 80% confluence, cells labeled with light amino acid were treated with 1 μg/mL ephrin-B2/Fc for 15 h, whereas cells labeled with heavy amino acid were treated with PBS for 15 h.

### Peptide preparation

Cells were lysed in urea lysis buffer (8 M urea in 0.1 M Tris-HCl, pH 8.5) containing protease and phosphatase inhibitors. Protein concentration was determined by Bradford assay. Equal amounts (125 μg) of light or heavy-isotope-labeled proteins were mixed and precipitated in pre-cooled acetone (-20 °C) for at least 1 h. Depending on different applications, precipitated protein was handled through the following steps:

#### Differentially expressed global protein sample

Precipitated proteins were dissolved in radioimmunoprecipitation assay (RIPA) lysis buffer and separated by sodium dodecyl sulfate polyacrylamide gel electrophoresis (SDS-PAGE) after centrifugation at 3,000 × g for 10 min. Gel was equally cut off into nine pieces on the basis of protein molecular weight. Each gel piece was reduced using DL-Dithiothreitol (DTT), alkalized with iodoacetamide (IAM), and in-gel digested with modified trypsin (Promega). Peptides were extracted, desalted with SepPak C18 (Waters, USA), and stored at -80 °C.

#### Differentially expressed phosphorylated protein sample

After centrifugation at 3,000 × g for 10 min, precipitated proteins were dissolved in 200 μL of urea lysis buffer containing 0.05 M DTT for 45 min and then mixed with 300 μL of iodoacetamide (IAM) solution (0.05 M IAM in urea lysis buffer). Mixture was incubated in the dark for 20 min and centrifuged in an ultrafiltration tube (Millipore, 30 K) at 14,000 × g for 15 min. Up to 300 μL of ammonium bicarbonate (ABC) solution (0.05 M NH_4_HCO_3_ in H_2_O) was added into ultrafiltration tube and then subsequently centrifuged at 14,000 × g for 15 min. This step was repeated twice. Trypsin (1:50) was added to protein samples, and resulting mixture was added to 100 μL of ABC solution. The sample was vibrated and incubated at 37 °C for 4–18 h. Up to 50 μL of H_2_O was added, and the tube was centrifuged in a new collector at 4 °C for 15 min. This step was repeated, and liquid effluents containing peptides were combined. Subsequently, 1% formic acid (final concentration) was added to effluents, which were desalted with SepPak C18. Peptides were dried and stored at -20 or -80 °C. Phosphorylated peptide enrichment was performed using titanium dioxide affinity chromatography for phosphorylated protein analysis.

### Peptide detection and data analysis

Dried peptides were dissolved in 0.1% formic acid/2% acetonitrile and analyzed on AB Sciex Triple TOF 5600 mass spectrometer (MS) system. Acquired raw data files (.wiff) were searched using Protein Pilot software V.4.2 (AB Sciex, USA) against protein sequence database. Finally, obtained data were uploaded to IPA (https://www.qiagenbioinformatics.com/products/ingenuity-pathway-analysis/) for bioinformatics analysis. As SILAC is a highly accurate quantitative method, cutoff for fold change was set to 1.50, and *p*-value was less than 0.05. This study employed global functional analyses, network analyses, and canonical pathway analyses.

For differentially expressed global protein samples, peptide sample of each gel piece was individually analyzed on MS. After searching against protein sequence database, nine sets of data were merged. Above experimental process was repeated thrice; data were merged and then uploaded to IPA for analysis.

### Cell proliferation assay

HUVECs were seeded in 96-well plate at a density of 3,000 cells per well. After overnight serum starvation, cells were incubated in DMEM containing 2% FBS and different ephrin-B2/Fc concentrations at 37 °C and 5% CO_2_. Cell proliferation was measured using CCK-8 following manufacturer's instructions at indicated times (1, 3, 5, and 7 days). PBS was used in control group. Eight repetitive wells were set for each group.

### Cell cycle analysis

After 24 h of serum starvation, HUVECs were cultured in DMEM containing 2% FBS and 1 μg/mL ephrin-B2/Fc at 37 °C for 24 h. Cells were then harvested and fixed in 70% pre-cooled ethanol at 4 °C. After incubation with 100 mg/L RNase A at 37 °C for 30 min, 50 mg/L propidium iodide was added into suspended cells in the dark for 30 min at room temperature. More than 10,000 cells in each sample were analyzed by flow cytometry (Bacton Dickson Co.), and data were analyzed using MultiCycle for Windows 32-bit software.

### Wound healing assay

HUVECs were seeded on six-well plates and grown to 100% confluence. After 12 h of serum starvation, consistently shaped wounds were made using a sterile 200 μL pipette tip across bottom surfaces of each well, creating cell-free areas. Cultures were gently washed with PBS to remove loose cells. Cells were cultured in serum-free medium containing different ephrin-B2/Fc concentrations or PBS for 24 h, fixed in 4% paraformaldehyde for 20 min, and then stained with crystal violet for 30 min at room temperature. Cell images were captured under an optical microscope. Six different scraped areas of each well were randomly selected, and cell numbers were counted.

### Transwell migration assay

Migration assay was performed using Transwell chambers (8 μm in pore size, Corning). A total of 400 μL serum-free DMEM containing different ephrin-B2/Fc concentrations was added to the lower chamber, whereas 3.5 × 10^4^ cells in 200 μL of DMEM were planted onto the upper chamber. Cells were cultured for 6 h at 37 °C under humidified 5% CO_2_ atmosphere. Cells in upper chamber were tenderly removed using wet cotton swabs, whereas lower cells were fixed with 4% paraformaldehyde for 20 min and stained with crystal violet for 30 min. Micrographs of six fields were randomly obtained under an inverted microscope, and cells were counted. Three independent experiments were performed thrice.

### Cell survival ratios assay

HUVECs were cultured in serum-free medium containing different concentrations of H_2_O_2_ for 4 h. Survival ratios were tested by CCK-8 assay according to instruction. Next, cells cultured in serum-free medium were incubated within PBS or different concentrations (0.1, 0.3, and 1 μg/mL) of ephrin-B2/Fc for 2 h, then 0.3 mmol/L H_2_O_2_ was added for another 4 h. Cell survival ratios were also tested using CCK-8 kit.

### Western blot

Cells were treated with different ephrin-B2/Fc concentrations for 0, 5, 20, and 60 min and 3 h and were lysed with RIPA buffer supplemented with protease inhibitors. Protein concentrations were measured using bicinchoninic acid (BCA) method. Equal amounts of proteins were separated with SDS-PAGE and then electrophoretically transferred to nitrocellulose membranes. Membranes were blocked in 5% bovine serum albumin for 1 h at room temperature and then incubated overnight with primary antibodies diluted in Tris-buffered saline with Tween-20 (TBS-T) at 4 °C. All primary antibodies were diluted at 1:1000 except for anti-c-Myc antibody, which was diluted at ratio of 1:800. Membranes were washed thrice with TBS-T buffer and incubated with corresponding secondary antibodies diluted (1:5000) in TBS-T. Bound antibodies were detected through chemiluminescence (Pierce). Quantity One (version 4.4) was used for quantitative analysis.

### Data analysis and statistics

All assays were performed in three separate experiments conducted in triplicate. Results were reported as mean ± standard error of the mean (SEM) of three independent experiments. Comparisons were performed using two-tailed paired Student's t-test. *p < 0.05, **p < 0.01, and ***p < 0.001.

## SUPPLEMENTARY MATERIALS FIGURES AND TABLES




