# Supplementary material for: Ephrin-B2/Fc promotes proliferation and migration, and suppresses apoptosis in human umbilical vein endothelial cells
Source: Oncotarget. 2017 Apr 20;8(25):41348–63. doi: 10.18632/oncotarget.17298 (PMC5522204; doi:10.18632/oncotarget.17298)
Supplement: Supplementary file 1 [file oncotarget-08-41348-s001.pdf]

Ephrin-B2/Fc promotes proliferation and migration, and suppresses apoptosis in human umbilical vein endothelial cells

SUPPLEMENTARY MATERIALS

SUPPLEMENTARY FIGURES AND TABLES

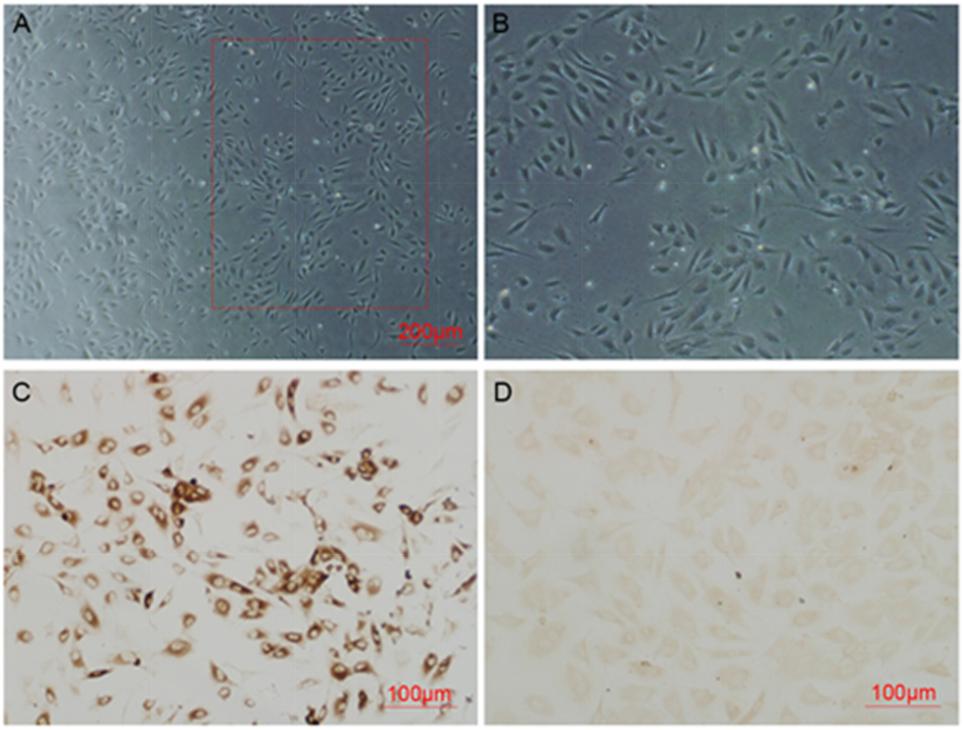

Supplementary Figure 1: Morphology and immunohistochemical identification of Human umbilical vein endothelial cells. **(A)** Observation of HUVECs extracted from umbilical cords by microscope. Scar bar = 200 μm. **(B)** Enlargement of the red box region from A. **(C)** and **(D)** Expression of human factor VIII related antigen detected by Immunohistochemical. Scar bar = 100 μm. HUVECs were cultured on sterile coverslips, fixed with solution containing acetone and ethanol (5: 4), blocked with 5% BSA at room temperature for 1h, and then incubated with Rabbit anti human factor VIII related antigen antibody **(C)** or PBS at 4 °C overnight.

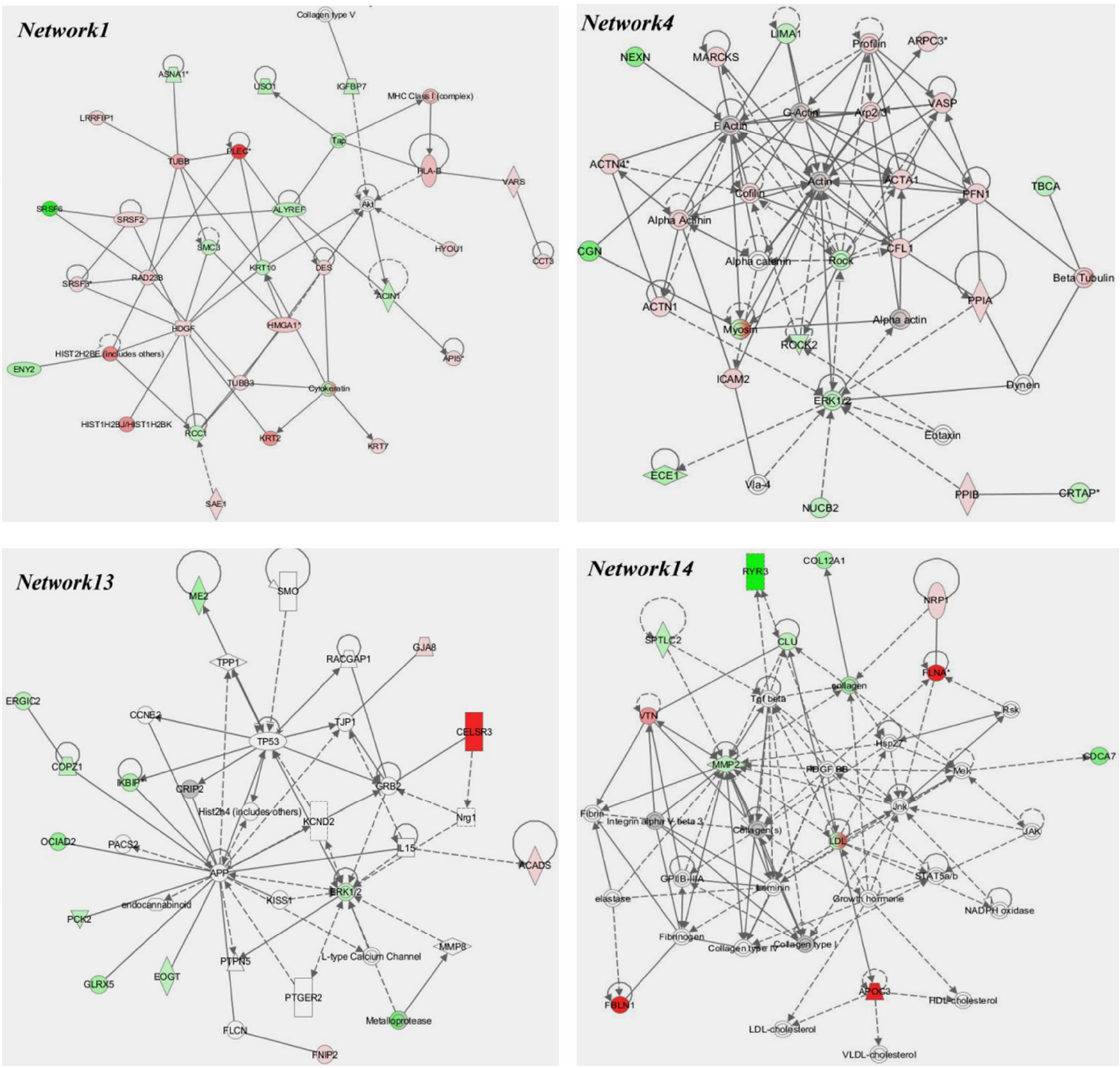

Supplementary Figure 2: Interaction networks with high significance that differentially expressed global proteins participated in. The biological relationship between two nodes was represented as an edge (line). The intensity of the node color indicated the degree of up-regulation (red) or down-regulation (green). The nodes without color represent the un-identified protein.

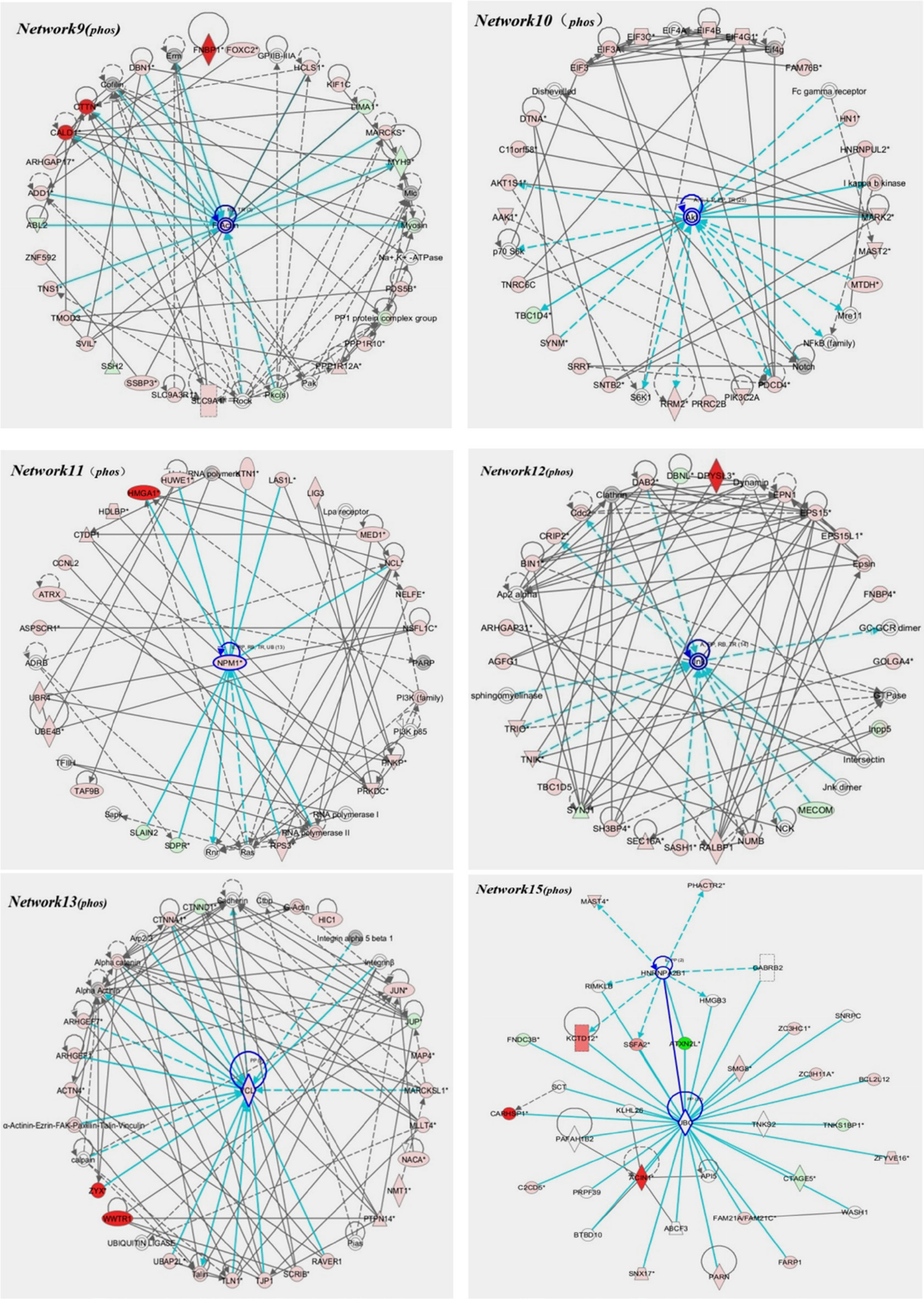

Supplementary Figure 3: Interaction networks with high significance that differentially expressed phosphorylated proteins participated in. The biological relationship between two nodes was represented as an edge (line). The intensity of the node color indicated the degree of up-regulation (red) or down-regulation (green). The nodes without color represent the un-identified protein.

Supplementary Table 1: Interaction networks with high significance that differentially expressed global proteins participated in and their associated functions

ID	Score	Focus Molecules	Top Diseases and Functions
1	58	30	Cell Death and Survival, Cancer, Dermatological Diseases and Conditions
4	30	19	Cellular Assembly and Organization, Cell Cycle, Cell Morphology
13	14	12	Cell-To-Cell Signaling and Interaction, Cellular Growth and Proliferation,
14	13	11	Cellular Movement, Cancer, Tumor Morphology

Supplementary Table 2: Interaction networks with high significance that differentially expressed phosphorylated proteins participated in and their associated functions

ID	Score	Focus Molecules	Top Diseases and Functions
9	33	23	Cell Assembly and Organization, Cellular Function and Maintenance, Cellular Movement
10	31	23	Cancer, Organismal Injury and Abnormalities, Reproductive System Disease
11	30	22	Cellular Movement, Cellular Assembly and Organization
12	30	24	Cellular Assembly and Organization, Cellular Movement, Connective Tissue Disorders
13	27	20	Cell Morphology, Cellular Assembly and Organization, DNA Replication
15	25	19	Protein Synthesis, Cellular Development, Cellular Growth and Proliferation

Note: The networks in Table 1 and Table 2 were established by searching against Ingenuity Pathways Knowledge Base with differentially expressed global and phosphorylated proteins, respectively. Each network was limited to 35 proteins. Score ≥10. Networks were scored for the likelihood of finding the focus gene(s)/proteins in the given network. The high score indicates low probability.

Supplementary Table 3: Summary of significant cell functions associated with differentially expressed global proteins

See Supplementary File 1

Supplementary Table 4: Summary of significant cell functions associated with differentially expressed phosphorylated proteins

See Supplementary File 1

Supplementary Table 5: Summary of significant signaling pathways associated with differentially expressed global proteins

Ingenuity Canonical Pathways	-log(p-value)	Ratio	z-score	Molecules
eNOS Signaling	1.68E00	3.55E-02	2.236	HSPA2, HSPA8, HSPA5, HSP90AB1, HSP90AA1
PPARα/RXRα Activation	1.3E00	2.79E-02	1.342	AP2A2, MAPK1, HSP90AB1, HSP90AA1, GPD2
Regulation of Actin-based Motility by Rho	2.45E00	5.49E-02	1.000	CFL1, PFN1, ARPC3, MYL12A, ACTA1
Rho A Signaling	2.61E00	4.92E-02	0.816	CFL1, PFN1, ARPC3, MYL12A, ACTA1, ROCK2
NRF2-mediated Oxidative Stress Response	6.06E00	6.67E-02	-0.447	DNAJC3, USP14, DNAJA3, DNAJC5, DNAJC16, MAPK1, DNAJB11, EPHX1, ACTA1, PPIB, VCP, GSTK1
Axonal Guidance Signaling	5.11E00	3.93E-02	NaN	MMP2, ARPC3, TUBB3, TUBB, VASP, MYL12A, ROCK2, ROBO3, TUBB4B, TUBB2A, TUBA1A, CFL1, PFN1, NRP1, MAPK1, GNG12, ERAP2

Supplementary Table 6: Summary of significant signaling pathways associated with differentially expressed phosphorylated proteins

Ingenuity Canonical Pathways	-log(p-value)	Ratio	z-score	Molecules
Integrin Signaling	3.22E00	6.93E-02	3.464	PIK3C2A, TLN1, VCL, CTTN, PPP1R12A, PXN, PTK2, ZYX, BRAF, ARHGEF7, ACTB, MAP2K2, ACTN4, FNBP1
Signaling by Rho Family GTPases	4.56E00	7.69E-02	3.153	JUN, IQGAP1, PIK3C2A, PKN1, PPP1R12A, SLC9A1, PPP1R12C, PTK2, VIM, CDC42EP1, ARHGEF7, CLIP1, ACTB, ARHGEF1, MAP2K2, SEPT9, STMN1, FNBP1
Gα12/13 Signaling	2.56E00	7.69E-02	3.000	IKBKG, JUN, F2R, PIK3C2A, ARHGEF1, MAP2K2, PXN, MEF2D, PTK2
Agrin Interactions at Neuromuscular Junction	3.49E00	1.16E-01	2.646	JUN, EGFR, ARHGEF7, CTTN, ACTB, PXN, PTK2, DAG1
Telomerase Signaling	3.72E00	1.01E-01	2.449	EGFR, TERF2, PIK3C2A, PTGES3, ELF4, MAP2K2, HSP90AB1, HSP90AA1, HDAC7, TERF2IP
ERK5 Signaling	3.01E00	1.11E-01	2.449	ATF2, EGFR, MEF2D, YWHAE, ELK4, FOSL1, MAP3K3
IGF-1 Signaling	1.96E00	7.22E-02	2.449	JUN, PIK3C2A, MAP2K2, PXN, PTK2, YWHAE, PRKAR1A
Rac Signaling	1.33E00	5.77E-02	2.449	JUN, IQGAP1, PIK3C2A, MAP2K2, PTK2, ELK4
NGF Signaling	1.28E00	5.61E-02	2.449	IKBKG, ATF2, PIK3C2A, MAP2K2, TRIO, MAP3K3
Cholecystokinin/ Gastrin-mediated Signaling	3E00	8.91E-02	2.333	JUN, ATF2, EGFR, MAP2K2, PXN, PRKD1, MEF2D, PTK2, FNBP1
Paxillin Signaling	2.97E00	8.82E-02	2.333	PIK3C2A, ARHGEF7, TLN1, VCL, ACTB, PTPN12, PXN, ACTN4, PTK2
ERK/MAPK Signaling	2.56E00	6.42E-02	2.309	ATF2, BRAF, PPP1R10, PIK3C2A, TLN1, PPP1R12A, ELF4, MAP2K2, PPP1R7, PXN, PTK2, PRKAR1A
VEGF Signaling	3.33E00	9.89E-02	2.121	PIK3C2A, VCL, ACTB, MAP2K2, PXN, NOS3, ACTN4, PTK2, YWHAE
HGF Signaling	2.88E00	8.57E-02	2.121	JUN, ATF2, PIK3C2A, ELF4, MAP2K2, PXN, PRKD1, PTK2, MAP3K3
PI3K/AKT Signaling	1.91E00	6.5E-02	2.121	IKBKG, INPP5D, MAP2K2, NOS3, HSP90AB1, YWHAE, HSP90AA1, SYNJ1
GNRH Signaling	1.79E00	6.2E-02	2.121	JUN, ATF2, EGFR, MAP2K2, PRKD1, PTK2, MAP3K3, PRKAR1A
IL-8 Signaling	2.18E00	6.01E-02	2.111	IKBKG, JUN, IQGAP1, MAP4K4, EGFR, BRAF, PIK3C2A, MAP2K2, PRKD1, PTK2, FNBP1
4-1BB Signaling in T Lymphocytes	2.14E00	1.29E-01	2.000	IKBKG, JUN, ATF2, MAP2K2
