# Supplementary material for: Ephrin-B2/Fc promotes proliferation and migration, and suppresses apoptosis in human umbilical vein endothelial cells
Source: Oncotarget. 2017 Apr 20;8(25):41348–63. doi: 10.18632/oncotarget.17298 (PMC5522204; doi:10.18632/oncotarget.17298)
Supplement: Supplementary file 2 [file oncotarget-08-41348-s002.docx]

Supplemental Table 3 Summary of significant cell functions associated with differentially expressed global proteins.

| Category | Function Annotation | P-Value | Z-Score | State | Molecules | Molecules |
| --- | --- | --- | --- | --- | --- | --- |
| Cell Death and Survival | apoptosis | 1.51E-06 | -3.56 | Inhibited | 75 | ACAA2↑, ACIN1↓, ACTN4↑, ANXA2↑, AP2A2↓, API5↑, APOA1↑, APOC3↑, ASTN1↓, BASP1↑, CAST↑, CFL1↑, CLU↓, CUX1↓, DES↑, DIABLO, DNAJA3↓, DNAJC3↓, DNAJC5↑, ECE1, EIF3F↑, EPHX1↓, F2↑, FAU↑, FBLN1↑, FN1↑, FNIP2↑, FUS↑, GAPDH↑↑, HDGF↑, HK2↑, HLA-B↑, HMGA1↑, HMGA2↑, HMGB1↑, HSP90AA1↑, HSP90AB1↑, HSPA2↑, HSPA5↑, HSPA8↑, HYOU1↑, ICAM2↑, IGFBP7↓, IKBIP↓, ILF3↑, JUP↓, KIF14↓, LMNA↑, MAPK1↓, MMP2↓, NAPA↑, NRP1↑, NT5E↓, PARK7↑, PCMT1↓, PDLIM4↓, PKM↑, PPIA↑, PRAF2↓, PRDM5↑, RAD23B↑, ROCK2↓, RUVBL2↓, SEP15↓, SH3GLB1↓, SPTLC2↓, SRSF2↑, STAT1↓, STMN1↑↑, SUB1↑↑, VASP↑, VCP↑, VDAC2↓, VTN↑, YBX3↑ |
|  | cell death | 4.97E-08 | -3.54 | Inhibited | 94 | ACAA2↑, ACIN1↓, ACTN4↑, ANXA2↑, AP2A2↓, API5↑, APOA1↑, APOB↑, APOC3↑, ASTN1↓, ATP13A2↑, BASP1↑, CAST↑, CCT3↑, CFL1↑, CLU↓, CUX1↓, DES↑, DIABLO↓, DNAJA3↓, DNAJC3↓, DNAJC5↑, ECE1↓, EIF3F↑, EPHX1↓, F13A1↑, F2↑, FAU↑, FBLN1↑, FLNA↑, FN1↑, FNIP2↑, FUS↑, GAPDH↑↑, GBE1↑, HADHA↓, HDGF↑, HK2↑, HLA-B↑, HMGA1↑, HMGA2↑, HMGB1↑, HSP90AA1↑, HSP90AB1↑, HSPA2↑, HSPA5↑, HSPA8↑, HYOU1↑, ICAM2↑, IGFBP7↓, IKBIP↓, ILF3↑, JUP↓, KIF14↓, KRT10↓, KRT2↑, LMNA↑, MAPK1↓, MCM7↑, MMP2↓, MYH9↑, NAPA↑, NRP1↑, NT5E↓, NUP93↓, PARK7↑, PCMT1↓, PDLIM4↓, PKM↑, PLEC↑, PPIA↑, PPIB↑, PRAF2↓, PRDM5↑, PSMC1↑, PSMD2↑, RAD23B↑, ROCK2↓, RUVBL2↓, SEP15↓, SH3GLB1↓, SPTLC2↓, SRSF2↑, STAT1↓, STMN1↑↑, SUB1↑↑, TUBA1A↑, TUBB↑, TUBB3↑, VASP↑, VCP↑, VDAC2↓, VTN↑, YBX3↑↑ |
|  | necrosis | 1.57E-05 | -3.07 | Inhibited | 70 | ANXA2↑, AP2A2↓, API5↑, APOA1↑, APOB↑, APOC3↑, ASTN1↓, ATP13A2↑, CAST↑, CCT3↑, CLU↓, CUX1↓, DIABLO↓, DNAJA3↓, DNAJC5↑, EIF3F↑, EPHX1↓, F13A1↑, F2↑, FAU↑, FBLN1↑, FLNA↑, FN1↑, FUS↑, GAPDH↑↑, GBE1↑, HADHA↓, HDGF↑, HK2↑, HLA-B↑, HMGA1↑, HMGA2↑, HMGB1↑, HSP90AA1↑, HSP90AB1↑, HSPA5↑, HSPA8↑, HYOU1↑, IGFBP7↓, JUP↓, KIF14↓, LMNA↑, MAPK1↓, MCM7↑, MMP2↓, NAPA↑, NRP1↑, NT5E↓, NUP93↓, PARK7↑, PDLIM4↓, PKM↑, PLEC↑, PPIA↑, PRAF2↓, PRDM5↑, PSMD2↑, RAD23B↑, ROCK2↓, SH3GLB1↓, SPTLC2↓, SRSF2↑, STAT1↓, STMN1↑↑, TUBA1A↑, TUBB↑, TUBB3↑, VCP↑, VDAC2↓, VTN↑ |
|  | cell survival | 6.26E-03 | 2.882 | Activated | 35 | APOB↑, CLU↓, CUX1↓, DIABLO↓, EEF2↑, F2↑, FLNA↑, FN1↑, GJA8↑, HDGF↑, HMGA1↑, HMGA2↑, HMGB1↑, HSP90AB1↑, HSPA5↑, HYOU1↑, IGFBP7↓, JUP↓, LMNA↑, MAPK1↓, NRP1↑, PARK7↑, PKM↑, PPIA↑, SMC3↓, SNRPE↑, STAT1↓, STMN1↑↑, TUBB↑, TUBB3↑, TUBGCP6↓, UBQLN2↑, USP14↓, VCP↑, VTN↑ |
| Organismal Development | growth of organism | 2.26E-03 | -2.17 | Inhibited | 19 | APOA1↑, CUX1↓, DNAJC3↓, F2↑, GPD2↓, HMGA2↑, HMGB1↑, HSD17B4↓, HSP90AA1↑, HSP90AB1↑, HSPA5↑, LMNA↑, MAPK1↓, MAPRE1↑, PAPSS2↓, PCMT1↓, RAD23B↑, RPL29↑↑, TLN1↑ |
| Cellular Movement | cell movement | 6.05E-05 | 4.218 | Activated | 56 | ACTN4↑, ANXA2↑, APOA1↑, APOB↑, ASTN1↓, CAST↑, CFL1↑, CLU↓, CUX1↓, DNAJA3↓, F13A1↑, F2↑, FHL1↑, FLNA↑, FN1↑, GNG12↑, HDGF↑, HIST1H1T↓, HMGB1↑, HSP90AA1↑, HSP90AB1↑, HSPA5↑, ICAM2↑, JUP↓, KRT10↓, KRT2↑, LIMA1↓, LMNA↑, MAPK1↓, MAPRE1↑, MARCKS↑, MCM7↑, MMP2↓, MTCH2↓, MYH10↓, MYH9↑, NEXN↓, NRP1↑, NT5E↓, NUCB2↓, PARK7↑, PFN1↑, PKM↑, PLEC↑, PPIA↑, PPIB↑, ROBO3↑, ROCK2↓, STAT1↓, STMN1↑↑, TLN1↑, TMEM30A↑, TUBA1A↑, USP14↓, VASP↑, VTN↑ |
|  | migration of cells | 2.27E-05 | 3.982 | Activated | 53 | ACTN4↑, ANXA2↑, APOA1↑, APOB↑, ASTN1↓, CAST↑, CFL1↑, CLU↓, CUX1↓, DNAJA3↓, F13A1↑, F2↑, FHL1↑, FLNA↑, FN1↑, GNG12↑, HDGF↑, HMGB1↑, HSP90AA1↑, HSP90AB1↑, HSPA5↑, ICAM2↑, JUP↓, KRT10↓, KRT2↑, LIMA1↓, LMNA↑, MAPK1↓, MAPRE1↑, MARCKS↑, MCM7↑, MMP2↓, MYH10↓, MYH9↑, NEXN↓, NRP1↑, NT5E↓, NUCB2↓, PARK7↑, PFN1↑, PKM↑, PLEC↑, PPIA↑, PPIB↑, ROBO3↑, ROCK2↓, STAT1↓, STMN1↑↑, TLN1↑, TMEM30A↑, TUBA1A↑, VASP↑, VTN↑ |
| Cellular Growth and Proliferation | proliferation of epidermal cells | 7.32E-03 | 2.425 | Activated | 7 | CLU↓, FN1↑, IGFBP7↓, JUP↓, KRT10↓, KRT2↑, STMN1↑ |
|  | proliferation of connective tissue cells | 5.68E-03 | 2.188 | Activated | 18 | ANXA2↑, CLTC↑↑, F2↑, FBLN1↑, FN1↑, HDGF↑, IGFBP7↓, JUP↓, KRT10↓, KRT2↑, LIMA1↓, MAPK1↓, MMP2↓, RPL29↑↑, SMC3↓, STAT1↓, STMN1↑↑, VTN↑ |
| Gene Expression | binding of DNA | 1.97E-03 | 2.31 | Activated | 18 | ALYREF↓, CFL1↑, CLU↓, CUX1↓, F2↑, FN1↑, GAPDH↑↑, HMGA1↑, HMGB1↑, JUP↓, LMNA↑, MAPK1↓, PPIA↑, PPIB↑, SMC3↓, STAT1↓, SUB1↑↑, YBX3↑↑ |
|  | binding of protein binding site | 1.75E-03 | 2.295 | Activated | 12 | 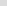CFL1↑, F2↑, FN1↑, HMGA1↑, HMGB1↑, JUP↓, LMNA↑, MAPK1↓, SMC3↓, STAT1↓, SUB1↑, YBX3↑ |
| Cellular Assembly and Organization | reorganization of cytoskeleton | 7.53E-04 | 2.194 | Activated | 9 | CFL1↑, F2↑, FLNA↑, FN1↑, HMGB1↑, JUP↓, MYH9↑, ROCK2↓, VTN↑ |
|  | organization of cytoplasm | 2.11E-04 | 2.036 | Activated | 39 | ACTN4↑, ATL3↑, BASP1↑, CAST↑, CFL1↑, CLTC↑, CLU↓, CNTNAP1↓, DES↑, DNAJA3↓, F13A1↑, F2↑, FLNA↑, FN1↑, GAPDH↑, HDGF↑, HMGB1↑, HSP90AA1↑, JUP↓, MAPK1↓, MAPRE1↑, MARCKS↑, MYH10↓, MYH9↑, NRP1↑, PDLIM7↓, PFN1↑, PKM↑, RCC1↓, ROBO3↑, ROCK2↓, STMN1↑, SURF4↓, TLN1↑, TUBB↑, TUBB3↑, TUBGCP6↓, VASP↑, VTN↑ |
| Cellular Function and Maintenance | organization of cytoskeleton | 3.59E-04 | 2.074 | Activated | 36 | ACTN4↑, BASP1, CAST↑, CFL1↑, CLU↓, CNTNAP1, DES, DNAJA3↓, F13A1↑, F2↑, FLNA↑, FN1↑, GAPDH↑, HDGF↑, HMGB1↑, HSP90AA1↑, JUP↓, MAPK1↓, MAPRE1↑, MARCKS↑, MYH10↓, MYH9↑, NRP1↑, PDLIM7, PFN1↑, PKM↑, RCC1, ROBO3↑, ROCK2↓, STMN1↑↑, TLN1↑, TUBB, TUBB3, TUBGCP6↓, VASP↑, VTN↑ |
| Cardiovascular System Development and Function | migration of endothelial cells | 2.07E-03 | 2.013 | Activated | 12 | ANXA2↑, FLNA↑, FN1↑, HMGB1↑, HSP90AB1↑, HSPA5↑, MAPK1↓, MARCKS↑, MMP2↓, NRP1↑, VASP↑, VTN↑ |
| Developmental Disorder | congenital anomaly of musculoskeletal system | 1.87E-03 | -2.34 | Inhibited | 20 | ACTA1↑, AHNAK↑, APOB↑, CAST↑, CFL1↑, ECE1↓, FBLN1↑, FHL1↑, FLNA↑, HSPA5↑, LMNA↑, MARCKS↑, PAPSS2↓, PLEC↑, RAD23B↑, SMCHD1↑, TMEM43↓, TUBGCP6↓, VCP↑, YBX3↑↑ |

**Supplemental Table 4** Summary of significant cell functions associated with differentially expressed phosphorylated proteins.

| **Diseases Or Functions Annotation** | **P-Value** | **State** | **Z-Score** | **Molecules** | **Molecules** |
| --- | --- | --- | --- | --- | --- |
| proliferation of cells | 5.43E-18 | Activated | 3.818 | 213 | ABCC4,ABL2,ACIN1,ACTB,ACTN4,AHNAK,AKAP12,AKT1S1,ARHGAP17,ARHGEF1,ARHGEF7,ARID1A,ASPSCR1,ATF2,BAG6,BCLAF1,BIN1,BRAF,BRIP1,BUB3,CACNA1D,CALD1,CAPRIN1,CAST,CCDC6,CCNL2,CDK1,CHD4,CHD8,CLIP1,CRTC1,CTNNA1,CTNND1,CTTN,CUL4B,CUX1,DAB2,DBN1,DDB2,DLC1,DNAJC2,DOCK4,DOT1L,DPYSL3,EGFR,EIF3A,EIF3C,EIF4B,EIF4G1,ELF4,ELK4,EPB41L3,EPN1,EPS15,EPS15L1,EZH2,F2R,FASN,FLNA,FNDC3B,FOSL1,FOXC2,G3BP1,GAPDH,HCLS1,HDAC7,HDGF,HIC1,HMGA1,HMGN1,HNRNPA1,HNRNPC,HNRNPK,HNRNPM,HNRNPU,HSP90AA1,HSP90AB1,HUWE1,IFI16,IGF2BP1,IKBKG,ILF3,INPP5D,IQGAP1,JUN,JUP,KMT2D,LARP1,LARP7,LAS1L,LIG1,LIMA1,LMNA,LMNB1,MAGED2,MAP1B,MAP2K2,MAP3K3,MAP4K4,MARCKS,MARCKSL1,MARK2,MAVS,MCM2,MECOM,MECP2,MED1,MKL1,MTA2,MTDH,MYBBP1A,MYH10,MYH9,NACA,NCL,NCOA3,NCOR1,NCOR2,NELFE,NES,NOL8,NOP2,NOP58,NOS3,NOSIP,NPM1,NUDCD3,NUMA1,NUMB,NUP214,NUP98,PA2G4,PCDH1,PDCD4,PDE4D,PDS5B,PEA15,PEAK1,PGRMC1,PIK3C2A,PKN1,PLEC,PML,PNKP,PNN,PPP1R10,PPP1R12A,PRKAA1,PRKAR1A,PRKD1,PRKDC,PTGES3,PTK2,PTPN12,PTPN14,PXN,RALBP1,RASIP1,RBM10,REST,RRM2,RSL1D1,RTN4,SAFB,SCARF1,SCRIB,SEPT9,SH3BP4,SIRT1,SLC12A4,SLC12A6,SLC9A1,SLC9A3R1,SND1,SP110,SPTBN1,SRRT,SRSF6,SSBP3,STK24,STMN1,SUPV3L1,SYNM,TACC3,TAF9B,TCF3,TERF2,TFE3,TJAP1,TJP1,TJP2,TLN1,TMPO,TNIK,TOPORS,TP53BP1,TPR,TRIM28,TRIO,TRRAP,TUBA1A,TUBB4B,UBE4B,USP5,USP8,UTP20,VCL,VIM,WTAP,WWTR1,YBX1,ZBTB7A,ZYX |
| pericardial effusion | 3.24E-03 | Inhibited | -2.000 | 6 | F2R,HDAC7,MED1,TUBA1A,TUBB4B,UBE4B |
| cardiomyopathy | 5.74E-04 | Inhibited | -2.093 | 21 | BAG3,BIN1,CACNA1D,CAST,CTNNA1,DAG1,DTNA,FOSL1,IKBKG,JUP,LMNA,MYH10,MYH9,NOS3,PDE4D,PDLIM5,PRKD1,TLN1,TMPO,VCL,WWTR1 |
| development of cardiovascular system | 8.24E-05 | Activated | 2.398 | 60 | ADD1,ARID1A,ATF2,BCLAF1,BIN1,BRAF,CHD7,DAB2,DLC1,EGFR,ESAM,F2R,FLNA,FOXC2,GRK6,HDAC7,HMGA1,JUN,JUP,KAT7,LUZP1,MAP1S,MAP2K2,MAP3K3,MARCKSL1,MECOM,MED1,MKL1,MTDH,MYH10,NCOA3,NCOR2,NOS3,PIK3C2A,PLEC,PML,PRKAA1,PRKAR1A,PRKD1,PRKDC,PTK2,PTPN12,PXN,RAD23B,RASIP1,REST,ROBO4,SASH1,SEPT9,SIRT1,SP100,SPTBN1,TMOD3,UBE4B,UBR4,USP8,VCL,VIM,WTAP,YWHAE |
| angiogenesis | 2.81E-04 | Activated | 2.928 | 44 | ADD1,ATF2,BAG3,BIN1,BRAF,CHD7,EGFR,ESAM,F2R,FLNA,FOSL1,FOXC2,GRK6,HDAC7,HMGA1,JUN,JUP,MAP1S,MAP3K3,MARCKSL1,MED1,MKL1,MTDH,MYH10,MYH9,NCL,NCOA3,NOS3,PIK3C2A,PLEC,PML,PRKD1,PTK2,RASIP1,ROBO4,RTN4,SASH1,SIRT1,SP100,SPTBN1,UBE4B,VCL,VIM,YWHAE |
| development of blood vessel | 1.67E-03 | Activated | 2.120 | 46 | ADD1,BCLAF1,BIN1,BRAF,CHD7,DAB2,EGFR,ESAM,F2R,FLNA,FOXC2,GRK6,HDAC7,HMGA1,JUN,JUP,LUZP1,MAP1S,MAP3K3,MARCKSL1,MED1,MKL1,MTDH,MYH10,NCOA3,NOS3,PIK3C2A,PLEC,PML,PRKAA1,PRKD1,PRKDC,PTK2,RAD23B,RASIP1,ROBO4,SASH1,SEPT9,SIRT1,SP100,SPTBN1,UBE4B,VCL,VIM,WTAP,YWHAE |
| interphase | 7.37E-07 | Activated | 2.623 | 45 | ACIN1,AKAP12,ATF2,ATRX,BAG3,BRAF,CDK1,CTNND1,CUX1,DOT1L,DTD1,EGFR,ELF4,EZH2,FASN,FLNA,GORASP2,HMGA1,HMGN1,IFI16,JUN,LAS1L,LMNA,LRWD1,MTDH,NCOA3,NES,NPM1,PEA15,PML,POLR2A,PRKAA1,PRKDC,PTGES3,PTK2,RAD18,RCC1,SIRT1,SSH2,SYNM,TCF3,TFE3,TMPO,TP53BP1,USP8 |
| G1 phase | 1.97E-05 | Activated | 2.959 | 28 | ACIN1,AKAP12,BAG3,BRAF,CDK1,CUX1,DOT1L,DTD1,EGFR,ELF4,EZH2,FASN,HMGA1,IFI16,JUN,LAS1L,LMNA,LRWD1,NCOA3,PRKDC,PTGES3,PTK2,RCC1,SIRT1,SSH2,SYNM,TCF3,TMPO |
| cell survival | 2.76E-05 | Activated | 3.825 | 74 | ABL2,ARID1A,ATF2,ATRX,BAG3,BCLAF1,BRAF,BRIP1,CDK1,CHD4,CUX1,DAB2,EGFR,EIF3A,EIF3C,EIF4G1,ELF4,ERCC5,F2R,FLNA,FOSL1,HDGF,HMGA1,HMGN1,HNRNPU,HNRNPUL2,HSP90AB1,IKBKG,INPP5D,IQGAP1,JUN,JUP,KIF1C,LIG1,LIG3,LMNA,MECOM,MED1,MTDH,NOP58,NOS3,NOSIP,PDCD4,PEA15,PGRMC1,PIK3C2A,PML,PNKP,PRKAA1,PRKAR1A,PRKD1,PRKDC,PSMA3,PTK2,RAD18,REST,RRM2,RSF1,SETMAR,SIRT1,SKA3,SLC9A1,SMARCC2,SND1,STMN1,SVIL,SYNM,TCF3,TP53BP1,TRIM28,USP8,VCL,VIM,YBX1 |
| cell death | 6.58E-13 | Inhibited | -2.938 | 185 | AAK1,ABCC4,ABL2,ACIN1,ACTB,ACTN4,ADD1,ADD3,AHCTF1,AKAP12,AKAP8,AKT1S1,ARHGEF1,ARHGEF7,ARID1A,ATF2,ATRX,BAG3,BAG6,BCL2L12,BCLAF1,BIN1,BRAF,CACNA1D,CANX,CAPRIN1,CAST,CCAR2,CCDC6,CCDC86,CCNL2,CDK1,CHD8,CTNNA1,CTNND1,CTTN,CUL4B,CUX1,DAB2,DAG1,DDB2,DLC1,DOT1L,DPF2,DPYSL3,EGFR,EIF3C,EIF4B,EIF4G1,EIF5B,ELF4,ERCC5,EZH2,F2R,FAM129B,FASN,FLNA,FOSL1,GAPDH,GFPT1,GRK6,HCLS1,HDGF,HIC1,HMGA1,HNRNPA1,HNRNPC,HNRNPK,HSP90AA1,HSP90AB1,HUWE1,IFI16,IGF2BP1,IKBKG,ILF3,INPP5D,JUN,JUP,KAT7,KIAA1468,KIF1C,KRT18,LIG1,LIG3,LMNA,LMNB1,MAP1B,MAP1S,MAP2K2,MAP3K3,MAP4,MAP4K4,MARK2,MAVS,MBOAT7,MCM2,MECOM,MECP2,MED1,MEF2D,MKL1,MTA2,MTDH,MYH9,NCL,NCOA3,NCOR2,NOS3,NPM1,NUDCD3,NUMA1,NUMB,PA2G4,PDCD4,PDE4D,PEA15,PGRMC1,PIEZO1,PKN1,PLEC,PML,PNKP,POLR2A,PPP1R10,PRKAA1,PRKAR1A,PRKD1,PRKDC,PTGES3,PTK2,PXN,RAD18,RAD23B,RALBP1,RANBP2,RBM25,REST,RPL27A,RPS3,RRM2,RSL1D1,RTN1,RTN4,SAFB,SCRIB,SEPN1,SIRT1,SKA3,SLC12A6,SLC9A1,SND1,SON,SP110,SPTBN1,SRRT,SRSF6,STK24,STMN1,SUPV3L1,SVIL,TACC3,TAF9B,TBL1XR1,TCF3,TERF2,TJP2,TMEM57,TMX1,TOPORS,TP53BP1,TPR,TRIM28,TUBA1A,UBE4B,UBR4,VAMP3,VCL,VIM,WTAP,YBX1,YWHAE,ZBTB7A,ZC3HC1,ZFYVE16,ZYX |
| apoptosis | 7.07E-13 | Inhibited | -2.329 | 155 | ABL2,ACIN1,ACTN4,AHCTF1,AKAP12,AKAP8,AKT1S1,ARHGEF7,ATF2,ATRX,BAG3,BAG6,BCL2L12,BCLAF1,BIN1,BRAF,CANX,CAPRIN1,CAST,CCAR2,CCDC6,CCDC86,CCNL2,CDK1,CHD8,CTNNA1,CTNND1,CTTN,CUL4B,CUX1,DAB2,DAG1,DDB2,DLC1,DOT1L,DPF2,EGFR,EIF3C,EIF4B,EIF4G1,ELF4,ERCC5,EZH2,F2R,FAM129B,FASN,FLNA,FOSL1,GAPDH,GFPT1,HCLS1,HDGF,HIC1,HMGA1,HNRNPA1,HNRNPC,HNRNPK,HSP90AA1,HSP90AB1,HUWE1,IFI16,IGF2BP1,IKBKG,ILF3,INPP5D,JUN,JUP,KAT7,KIF1C,KRT18,LIG1,LMNA,LMNB1,MAP1B,MAP1S,MAP2K2,MAP3K3,MAP4,MAP4K4,MAVS,MBOAT7,MCM2,MECOM,MECP2,MED1,MEF2D,MKL1,MTA2,MTDH,NCL,NCOA3,NCOR2,NOS3,NPM1,NUMA1,NUMB,PA2G4,PDCD4,PDE4D,PEA15,PKN1,PML,PNKP,POLR2A,PPP1R10,PRKAA1,PRKAR1A,PRKD1,PRKDC,PTK2,PXN,RAD23B,RALBP1,RBM25,REST,RPS3,RRM2,RSL1D1,RTN1,RTN4,SAFB,SCRIB,SEPN1,SIRT1,SLC9A1,SND1,SON,SP110,SPTBN1,SRRT,STK24,STMN1,SUPV3L1,TACC3,TAF9B,TBL1XR1,TCF3,TERF2,TJP2,TMX1,TOPORS,TP53BP1,TPR,TRIM28,TUBA1A,UBE4B,UBR4,VCL,VIM,WTAP,YBX1,YWHAE,ZBTB7A,ZC3HC1,ZFYVE16 |
| cell viability | 1.18E-04 | Activated | 3.916 | 67 | ABL2,ARID1A,ATF2,ATRX,BAG3,BCLAF1,BRAF,BRIP1,CDK1,CHD4,CUX1,DAB2,EGFR,EIF3A,EIF3C,EIF4G1,ELF4,F2R,FLNA,FOSL1,HDGF,HMGA1,HMGN1,HNRNPU,HNRNPUL2,HSP90AB1,IKBKG,INPP5D,IQGAP1,JUN,KIF1C,LIG1,LIG3,LMNA,MECOM,MED1,MTDH,NOP58,PDCD4,PGRMC1,PIK3C2A,PML,PNKP,PRKAA1,PRKAR1A,PRKD1,PRKDC,PSMA3,PTK2,RAD18,REST,RRM2,RSF1,SETMAR,SIRT1,SKA3,SLC9A1,SMARCC2,SND1,STMN1,SVIL,SYNM,TCF3,TP53BP1,TRIM28,USP8,YBX1 |
| cell movement | 5.76E-12 | Activated | 3.118 | 128 | ABCC4,ABL2,ACTB,ACTN4,ADD1,AKAP11,AKAP12,ARHGAP31,ARHGEF1,ARHGEF7,ATF2,BAG3,BRAF,CAST,CDC42EP1,CDK1,CRIP2,CTNNA1,CTNND1,CTTN,CUX1,DAB2,DAG1,DBN1,DDB2,DLC1,DOCK4,DPYSL3,EGFR,EIF3A,ELF4,ESAM,EZH2,F2R,FASN,FLNA,FNDC3B,FOSL1,FOXC2,GRK6,HCLS1,HDGF,HIC1,HNRNPK,HSP90AA1,HSP90AB1,IGF2BP1,IKBKG,ILF3,INPP5D,IQGAP1,JUN,JUP,KIF1C,KMT2D,LIMA1,LMNA,LMNB1,LMO7,MAP1B,MAP2K2,MAP4K4,MARCKS,MARCKSL1,MARK2,MAVS,MBOAT7,MCM2,MED1,MKL1,MLLT4,MTDH,MYH10,MYH9,MYO9B,NACA,NCL,NCOA3,NES,NOS3,NPM1,NUMB,PA2G4,PDCD4,PEAK1,PHACTR4,PIK3C2A,PLEC,PML,PRKAR1A,PRKD1,PTK2,PTPN12,PTPN14,PXN,RALBP1,REST,ROBO4,RTN4,SASH1,SEPT9,SH3BP4,SIRT1,SLC12A6,SLC9A1,SLC9A3R1,SNX17,SORBS3,SP100,STK24,STMN1,SYNM,TCF3,TERF2,TJP1,TLN1,TMOD3,TNS1,TPR,TRIO,TUBA1A,VCL,VIM,WWTR1,YBX1,YWHAE,ZBTB7A,ZYX |
| migration of cells | 2.04E-11 | Activated | 3.194 | 117 | ABCC4,ABL2,ACTB,ACTN4,ADD1,AKAP11,ARHGAP31,ARHGEF1,ARHGEF7,ATF2,BAG3,BRAF,CAST,CDC42EP1,CDK1,CRIP2,CTNNA1,CTTN,CUX1,DAB2,DAG1,DBN1,DLC1,DOCK4,DPYSL3,EGFR,ELF4,ESAM,EZH2,F2R,FASN,FLNA,FNDC3B,FOSL1,FOXC2,GRK6,HCLS1,HDGF,HIC1,HNRNPK,HSP90AA1,HSP90AB1,IKBKG,ILF3,INPP5D,IQGAP1,JUN,JUP,KMT2D,LIMA1,LMNA,LMNB1,LMO7,MAP1B,MAP2K2,MAP4K4,MARCKS,MARCKSL1,MARK2,MAVS,MBOAT7,MCM2,MKL1,MLLT4,MTDH,MYH10,MYH9,MYO9B,NACA,NCL,NCOA3,NES,NOS3,NPM1,NUMB,PA2G4,PDCD4,PEAK1,PHACTR4,PIK3C2A,PLEC,PML,PRKAR1A,PRKD1,PTK2,PTPN12,PTPN14,PXN,RALBP1,REST,ROBO4,RTN4,SASH1,SH3BP4,SIRT1,SLC9A1,SLC9A3R1,SNX17,SORBS3,SP100,STK24,STMN1,SYNM,TCF3,TERF2,TLN1,TMOD3,TNS1,TRIO,TUBA1A,VCL,VIM,WWTR1,YBX1,YWHAE,ZBTB7A,ZYX |
| Edema | 1.18E-04 | Inhibited | -2.473 | 24 | ABL2,BAG6,DBNL,DTNA,F2R,FLNA,FOXC2,IKBKG,INPP5D,JUN,MECOM,MKL1,NCOR1,NOS3,NUMB,PIEZO1,PRKAR1A,PTPN14,RAD23B,RASIP1,SIRT1,SLC12A4,UBE4B,YBX1 |
| repair of DNA | 1.45E-08 | Activated | 2.163 | 28 | ATF2,BCLAF1,BRIP1,CDK1,DDB2,EGFR,EIF3A,ERCC5,HMGA1,HMGN1,HNRNPU,HUWE1,JUN,LIG1,LIG3,NPM1,PNKP,PRKDC,RAD18,RAD23B,SETMAR,SIRT1,SMARCC2,TERF2,TP53BP1,TRIM28,TRRAP,YBX1 |
